# Development of a modularized two-step (M2S) chromosome integration technique for integration of multiple transcription units in *Saccharomyces cerevisiae*

**DOI:** 10.1186/s13068-016-0645-4

**Published:** 2016-10-28

**Authors:** Siwei Li, Wentao Ding, Xueli Zhang, Huifeng Jiang, Changhao Bi

**Affiliations:** Key Laboratory of Systems Microbial Biotechnology, Tianjin Institute of Industrial Biotechnology, Chinese Academy of Sciences, 32 West 7th Ave, Tianjin Airport Economic Park, Tianjin, 300308 China

**Keywords:** Chromosome integration, Transcription unit, Module, DNA assembly

## Abstract

**Background:**

*Saccharomyces cerevisiae* has already been used for heterologous production of fuel chemicals and valuable natural products. The establishment of complicated heterologous biosynthetic pathways in *S. cerevisiae* became the research focus of Synthetic Biology and Metabolic Engineering. Thus, simple and efficient genomic integration techniques of large number of transcription units are demanded urgently.

**Results:**

An efficient DNA assembly and chromosomal integration method was created by combining homologous recombination (HR) in *S. cerevisiae* and Golden Gate DNA assembly method, designated as modularized two-step (M2S) technique. Two major assembly steps are performed consecutively to integrate multiple transcription units simultaneously. In Step 1, Modularized scaffold containing a head-to-head promoter module and a pair of terminators was assembled with two genes. Thus, two transcription units were assembled with Golden Gate method into one scaffold in one reaction. In Step 2, the two transcription units were mixed with modules of selective markers and integration sites and transformed into *S. cerevisiae* for assembly and integration. In both steps, universal primers were designed for identification of correct clones. Establishment of a functional β-carotene biosynthetic pathway in *S. cerevisiae* within 5 days demonstrated high efficiency of this method, and a 10-transcriptional-unit pathway integration illustrated the capacity of this method.

**Conclusions:**

Modular design of transcription units and integration elements simplified assembly and integration procedure, and eliminated frequent designing and synthesis of DNA fragments in previous methods. Also, by assembling most parts in Step 1 in vitro, the number of DNA cassettes for homologous integration in Step 2 was significantly reduced. Thus, high assembly efficiency, high integration capacity, and low error rate were achieved.

**Electronic supplementary material:**

The online version of this article (doi:10.1186/s13068-016-0645-4) contains supplementary material, which is available to authorized users.

## Background

*Saccharomyces cerevisiae* is a prominent model organism in the field of Synthetic Biology and Metabolic Engineering. It has already been used for heterologous production of fuel chemicals and valuable natural compounds, such as artemisinin [1, 2], taxol [3], ginsenosides [4], and tanshinones [5, 6]. More and more researchers are dedicated to establish complex heterologous biosynthetic pathways in *S. cerevisiae* [2, 7, 8]. Genes of the pathways need to be integrated into *S. cerevisiae* chromosome with regulation elements, such as promoters and terminators, in the form of transcription units [9]. Thus, to establish a functional heterologous pathway with multiple genes, many DNA parts need to be integrated into the chromosome. Fast and convenient yeast genomic integration techniques are demanded urgently to meet the development of complex yeast engineering.

Based on the high efficiency of homologous recombination (HR) in *S. cerevisiae*, genomic modification of yeast mainly relies on homologous recombination in vivo. One of the most mature methods was developed by Huimin Zhao’s group [10, 11], whose DNA assembler method is able to assemble multiple DNA cassettes in *S. cerevisiae*, either on a plasmid or on a chromosome. To demonstrate their technique, zeaxanthin and aureothin biosynthetic pathways were successfully constructed and proved to be functional [10, 12, 13]. This technique allows assembly of multiple DNA fragments in a single-step fashion, which is efficient and time-saving compared with traditional restriction enzyme-dependent cut-and-paste manipulation method; therefore, it has been widely applied to DNA assembly and strain engineering. However, due to the nature of homologous recombination, assembly efficiency significantly decreases when the number of DNA parts increases [14, 15]. Meanwhile, error rate of assembly (proportion of incorrectly assembled constructs) increases with more DNA parts to be assembled [12, 16]. In addition, frequent designing and synthesis of the overlap sequences are required for the DNA assembler method, making the integration process laborious and costly. A novel technique is desired to overcome these problems.

**Fig. 1:**
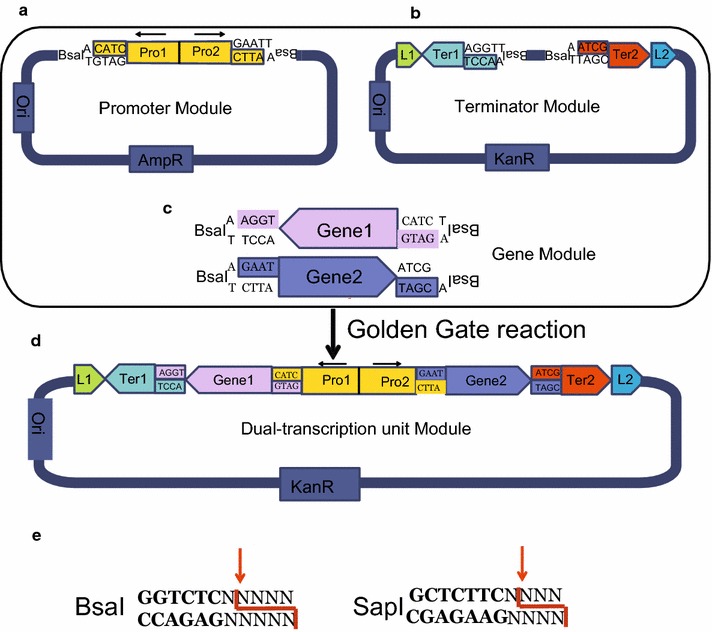
Modular strategy and construction of Step 1 assembly procedure. **a** Structure of head-to-head promoter module; **b** Structure of terminator module for Step 1 assembly; **c** Structure of modularized gene coding sequence for Step 1 assembly; **d** Structure of the two-transcription-unit module from Step 1 assembly; **e** Digestion characteristic of Type IIs restriction enzymes. *Letter N* represents a random base; and *colored region* represents remaining DNA fragments by digestion of enzyme

Golden Gate assembly method as a rapid cloning strategy relies on Type IIs restriction enzymes, which is employed to assemble several DNA fragments into a vector simultaneously [17–19]. The type IIs restriction enzymes are able to cleave DNA fragments outside of their recognition site, resulting in 4 nt or 3nt overhangs (Fig. 1b). With rational design of the cleavage sites, two or more digested fragments can be ligated to form a seamless product without restriction recognition sites [20], which simplifies in vitro gene-cloning procedure. Similar as all the DNA assembly techniques, Golden Gate also suffers decreased efficiency as assembly parts increase.

In this work, we exploit an efficient DNA assembly and chromosomal integration method by combining Golden Gate method and yeast HR with modularized designed parts strategy. In this method, functional parts, including promoters, terminators, and gene coding sequences, were equipped with fixed terminal sequence for Golden Gate assembly. However, the assembled transcription units along with readymade selective markers and DNA homologous arms were integrated in pre-selected specific locus of chromosome. These modularized parts were used for integration of different genes, with which in vitro assembly step only required the preparation of cloning genes with specific adapter sequences. Two transcription units were designed to be assembled in one Golden Gate reaction with the specific design of paired head-to-head promoters [21, 22]. In the following step, assembled DNA fragments in step one were integrated into yeast chromosome also with readymade modularized parts, including selective markers and integration homologous arms in one step manner. In this work, we demonstrated application of this fast and easy method by integration of a four-gene β-carotene biosynthetic pathway into *S. cerevisiae* within 5 days. In addition, libraries of modularized promoters and terminators and scaffolds were constructed to enable integrations of more genes simultaneously. This novel technique has a great potential to be widely adopted in Synthetic Biology and Metabolic Engineering research for yeast species.

## Results and discussion

### Design and construction of modular parts for dual-transcription unit assembly (Step 1)

A functional transcription unit in *S. cerevisiae* includes at least a promoter (Pro), a gene coding sequence (Gene), and a terminator (Ter). For direct and simple assembly of transcription units, we have designed a three-module strategy as shown in Fig. 1. Each modular part was attached with dedicated adapter sequence for Golden Gate assembly. Two promoters were head-to-head fused with adapters and Type IIs restriction enzyme sites flanked away at the ends of this part (Fig. 1a). Accordingly, genes were PCR amplified to equip specific adapter sequences on both sides to connect with promoter and terminator parts (Fig. 1c). Two terminators were inserted into scaffold plasmid of Step 1 assembly, with dedicated homologous sequences (L1 and L2) lying on both sides of them, which were designed for assembly with modules in Step 2. Two back-to-back Type IIS restriction enzyme sites with protection sequence were inserted between the two terminators for assembly with other modules in Step 1 (Fig. 1b). For assembly reaction of the dual-transcription unit in Step 1, the previously mentioned three parts were digested with Type IIS restriction enzyme, such as BsaI or SapI, to expose the designed complementary DNA sticky ends. At last, the dual-transcription unit was formed by T4 DNA ligase with Golden Gate procedure.

For example, with BsaI as restriction enzyme (Fig. 1e), the cleavage site of BsaI was located behind its recognition sequence (5′-GGTCTC-3′). After digestion, a 4-bp overhang was exposed and recognition sequence was removed meanwhile. With ligation of these adapters, the product was formed as 5′ L1-Ter1-AGGT-Gene1-CATC-Pro1-Pro2-GTAC-Gene2-ATCG-Ter2-L2 3′ (Fig. 1d). Generally, yeast promoters extend 500–1500 bp upstream of the start codon, and terminators are defined as sequence 200–500 bp downstream of the stop codon. To obtain promoters and terminators to construct modular parts, we determined the boundary of them based on previous research of our lab and reports by other researchers. For easy storage and amplification, modular parts of Step 1 were constructed in the form of plasmids based on plasmid pMD-19T or pUC57. Type IIS restriction enzyme sites and adapter sequences were embedded in primers for application of coding sequences for assembly. P1, P2, until Pn were used to name promoter plasmids. Similarly, T1 to Tn were used to indicate terminator vectors, which were also the scaffolds for assembly of Step 1 parts. As illustrated in Table 2, five promoter plasmids and five terminator plasmids were constructed with ten promoters and ten terminators, the plasmid diagrams are shown in Additional file 1: Figures S1, S2. The promoters we employed in the method were the ones widely used in metabolic engineering of *S. cerevisiae*. Constitutive promoters with medium strength were mainly used in this work [23–25]. Terminators we selected were also common terminators that are reported and often used by other researchers. This set of parts allowed up to ten transcription units to be assembled into yeast genome simultaneously. Modular parts in these plasmids can be used repeatedly without change. To ligate with gene coding sequences, corresponding adapter sequences are inserted to both sides of them by PCR (adaptors for genes primers as shown in Table 1), which is the only necessary procedure to prepare genes for integration. Sequences of promoters and terminators are listed in Additional file 1: Tables S1, S2. Universal primers were designed to identify correct assembled plasmids (Additional file 1: Table S5).

**Table 1:** Standardized adaptor sequence of individual gene for Golden Gate

	BsaI	SapI	BsmBI
Gene1 forward primer	*GGTCTC* a GATG*	*GCTCTTC* **a GAT**	*CGTCTCA* **a GATG**
Gene1 reverse primer	*GGTCTC* a **AGGT**	*GCTCTTC* a **AGG**	*CGTCTCA* a **AGGT**
Gene2 forward primer	*GGTCTC* **a GAAT**	*GCTCTTC* **a GAA**	*CGTCTCA* **a GAAT**
Gene2 reverse primer	*GGTCTC* a **CGAT**	*GCTCTTC* a **CGA**	*CGTCTCA* a **CGAT**

*Italic 6–7 base sequences are recognition sites; Bold 4 or 3 base sequences are overhang sites. All sequences are written from 5′ to 3′

### Design and construction of modular parts for genomic integration (Step 2)

**Fig. 2:**
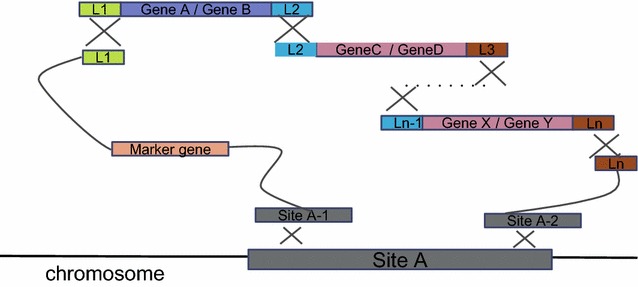
Step 2 assembly and integration based on in vivo homologous recombination*. L1* to *Ln* represent the homologous arms for recombination among fragments; Site A is the integration chromosomal locus. Modules with genes are products of Step 1 assembly; and modules with integration site sequences are readymade

Step 2 was to assemble multiple dual-transcription units into *S. cerevisiae* chromosome, which was based on homologous recombination of multiple fragments in vivo (Fig. 2). The key of the recombination process was the dedicated overlap regions between assembly parts, designated as L1, L2 to Ln. As illustrated in Fig. 2, 5′ terminal of first dual-transcription unit overlapped with the 3′ terminal of selection marker cassette, whose 5′ end sequence shared homologous sequence with a target site of chromosome. Similarly, every part was designed to overlap with adjacent ones, while the 3′ terminal of the last unit overlapped with the target site of chromosome for integration. To simplify integration procedure and improve efficiency of homologous recombination, these 150-bp overlap sequences were designed to have least homology with *S. cerevisiae* genome by sequence analysis. Deployed homologous sequences L1 to L6 are shown in Additional file 1: Table S3. Since different chromosome regions showed diversity of transcription levels, Jens Nielsen’s group characterized 20 different integration sites of the *S. cerevisiae* genome by inserting *lacZ* as a reporter gene under the control of two different promoters and determined expression levels through enzyme activity measurement. Seventeen of these sites are solo long terminal repeats (solo LTRs), none of them was located in close proximity to an open reading frame. Higher*β*-Galactosidase activity (The lacZ expression levels) of *S. cerevisiae* strains with integration sites of *YORWΔ17*, *YORWΔ22*, *YPRCΔ15,* and *YPRCτ3* was observed. Thus, we selected three sites *YORWΔ17*, *YORWΔ22*, and *YPRCΔ15* as our integration targets [26]. As common yeast chromosomal integration method, these modular parts were co-transformed into *S. cerevisiae* by electroporation. PCR analysis was used to verify correctly integrated strains with designed universal primers.

### Design of extra sets of adapters for increased compatibility of the M2S technique

In the process of Golden Gate assembly, presence of Type IIs restriction sites in gene coding sequence significantly decreases assembly efficiency. Alternative use of various Type IIs restriction enzymes, therefore restriction sites, is a strategy to avoid the existence of certain restriction sites in coding sequence and improve assembly efficiency. However, there are very limited Type IIs restriction enzymes available for Golden Gate assembly, namely BsaI, SapI, and BsmBI. Thus, the promoter parts and terminator parts were exhaustively designed to employ these enzyme sites with adaptor sequences accordingly (Table 1).

As listed in Table 2, we have constructed a series of promoter vectors and terminator vectors. Combinatory use of the vectors with various adapters guarantees assembly efficiency.

**Table 2:** Lists vectors used in this study

Plasmid	Genotypes	Sources
pMD-19T	Amp^+^	GENEWIZ
pUC57-Kan	Kan^+^	GENEWIZ
P1(pTDH3-pADH)	Amp^+^ promoter vector with promoters TDH3 and ADH1	This study
P2(pPGK1-pTEF2)	Amp^+^ promoter vector with promoters PGK1 and TEF2	This study
P3(pFBA-pHXT7)	Amp^+^ promoter vector with promoters FBA and HXT7	This study
P4(pTPI1-pTEF1)	Amp^+^ promoter vector with promoters TPI1 and TEF1	This study
P5(pPDC1-pPYK1)	Amp^+^ promoter vector with promoters PDC1 and PYK1	This study
T1-(tTPI1-PGIt)	Amp^+^ terminator vector, contains terminators TPI1 and PGI	This study
T2-(tADH1-tCYC1)	Amp^+^ terminator vector with terminators ADH1 and CYC1	This study
T3-(tFBA1-tPDC1)	Kan^+^ terminator vector with terminators FBA1 and PDC1	This study
T4-(tRPS2-tTDH1)	Kan^+^ terminator vector with terminators RPS2 and TDH1	This study
T5-(tCCW12-tRPL9A)	Kan^+^ terminator vector with terminators CCW12 and RPL9A	This study
pKna(15site-Ura3)	Kan^+^ selective marker and integration locus	This study
pKna(17site-His3)	Kan^+^ selective marker and integration locus	This study
pKna(22site-Leu2)	Kan^+^ selective marker and integration locus	This study

### Integration of a four-gene β-carotene biosynthetic pathway with M2S technique

**Fig. 3:**
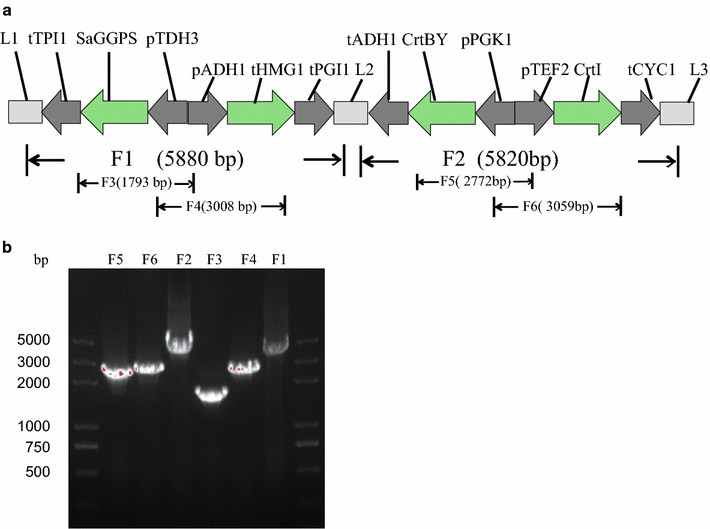
Structure and identification strategy of the assembled transcription unit modules of β-carotene biosynthetic pathway. **a** Structure and colony PCR strategy of the assembled transcription unit modules of β-carotene biosynthetic pathway, *F1* to *F6* indicate six PCRs designed to confirm correct assembly; **b** PCR gel picture for analysis of the transcription unit modules of β-carotene biosynthetic pathway

To illustrate the application of the M2S integration technique, β-carotene synthetic pathway was introduced into *S. cerevisiae.* This pathway includes carotenogenic genes *CrtBY* and *CrtI* from *Xanthophyllomyces dendrorhous*, whose expression enabled β-carotene production in yeast [27, 28]. In addition, overexpression of truncated 3-hydroxy-3-methylglutaryl-CoA reductase (tHMGR) from *S. cerevisiae* and GGPP synthase (GGPS) from *Sulfolobus acidocaldarius* could improve the supply of β-carotene intermediates, mevalonate, and GGPP [29]. With our M2S integration technique, two dual-transcription units, L1- *tTPI1*- *CrtBY*-*pTDH3*-*pADH1*- *CrtI*-*tPGI1*-L2 and L2- *tADH1*-*tHMG1*- *pPGK1* -*pTEF2*- *SaGGPS*-*tCYC1*-L3, were constructed as illustrated in previous text and Fig. 1. Each module, which was assembled in the form of a plasmid, included two transcription units and two homologous linkers. For colony PCR identification, F1 fragment was PCR amplified using the primer set L1F and L1R; F2 fragment with L2F and L3R, F3 fragment with pTDH3-F and SaGGPS-R, F4 fragment with pADH1-F and tHMG1-R, F5 fragment with pPGK1-F and CrtBY-R, and F6 fragment with pTEF2-F and CrtI-R (Fig. 3; Additional file 1: Table S5). In Step 1, the TU part construction and PCR verification took 2 days of work.

**Fig. 4:**
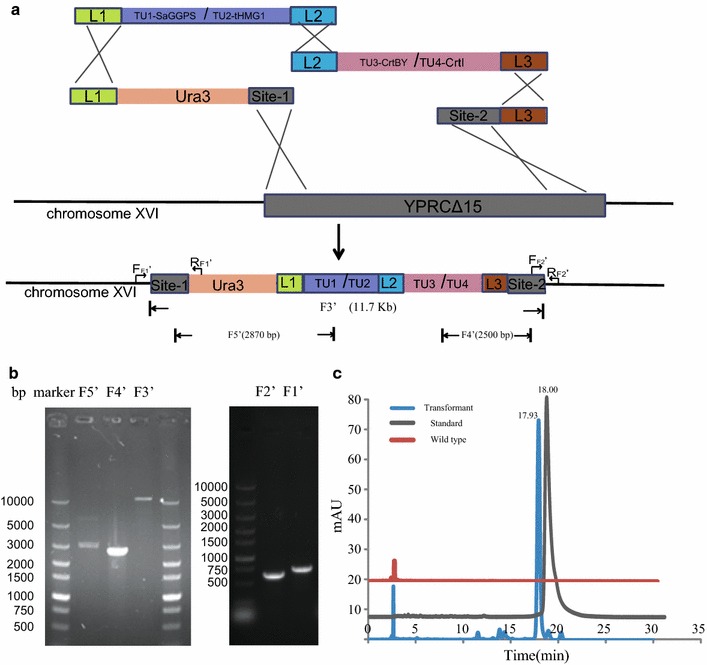
Structure and identification strategy of the assembled and integrated β-carotene biosynthetic pathway. **a** Structure and identification strategy of the assembled and integrated β-carotene biosynthetic pathway in *YPRCΔ15* site, F1’ to F5’ indicate five PCRs designed to confirm correct assembly and integration; **b** Gel picture of identification PCRs for assembly and integration. **c** HPLC analysis for β-carotene production

In Step 2, two readymade modules, site1-*Ura3*-L1 (Additional file 1: Figure S3) and L3-site2 (Additional file 1: Table S6), were used to integrate the two modules that constructed in Step 1. Gene YPF1(15 site, Additional file 1: Table S4) of yeast chromosome was used as target locus with Ura3 as selection marker. After electroporation and 2 days of incubation at 30 °C, hundreds of colonies appeared on the SC-Ura solid medium as shown in Additional file 1: Figure S4. The efficiency of transformation was calculated to be 2.751 × 10^3^ CFU/μg DNA. Colonies that appeared in orange color were confirmed to produce β-carotene by HPLC, while white colonies did not (Fig. 4c). Thus, orange colonies represented correctly assembled and integrated strains. The ratio of correct clones in total clones was calculated to be 58.75 ± 6.9%, based on color indication. Experiments were done in triplet to obtain the average.

In order to further confirm the exact DNA sequences of correctly assembled clones, 20 orange colonies were picked for genomic DNA extraction. The obtained genomic DNA was used as PCR template for confirmation. As shown in Fig. 3a, six PCRs were designed to confirm correct assembled sequences of the modules, with primers targeting overlap regions and promoter regions. These PCRs were same as the ones for TU part verification. All tested orange colonies had the six PCR products as expected on agarose gel (Fig. 3b). To identify correct integration of β-carotene pathway, F1’ fragment (about 680 bp) was PCR amplified using the primer set F_F1’_ and R_F1’_, F2’ fragment (about 640 bp) was PCR amplified using the primer set F_F2’_ and R_F2’_. Primers F_F1’_ and R_F2’_ were designed to anneal with DNA sequence flanking *YPRC*Δ15 site on *chromosome XVI*; R_F1’_ was designed to anneal with Ura3 fragment; and F_F2’_ was designed to anneal with site2. Fragment F3’ was PCR amplified with primer set Y15site1F and Y15site2R, resulting a PCR product of 11.7 Kb, which was the whole assembled β-carotene pathway cassette. F4’ fragment was obtained with 15site1 (134)-F and pTDH3 (273)-R, and F5’ fragment with pTEF2 (211)-F and 15site2 (316)-R. PCR product F1’ to F5’ indicated correct assembly and integration of the four fragments (dual-transcription units, L1-Uras-site1 and site2-L3) on yeast chromosome (Fig. 4).

### Integration of a ten-gene biosynthetic pathway with M2S technique

Five dual-transcription unit plasmids, L1-*tTPI1*-*G1*-*pTDH3*-*pADH1*-*G2*-*tPGI1*-L2, L2-*tADH1*-

*G3*-*pPGK1*-*pTEF2*-*G4*-*tCYC1*-L3, L3-*tFBA1*-*G5*-*pTDH3*-*pADH1*-*G6*-*tPDC1*-L4, L4- *tRPS2*-

**Fig. 5:**
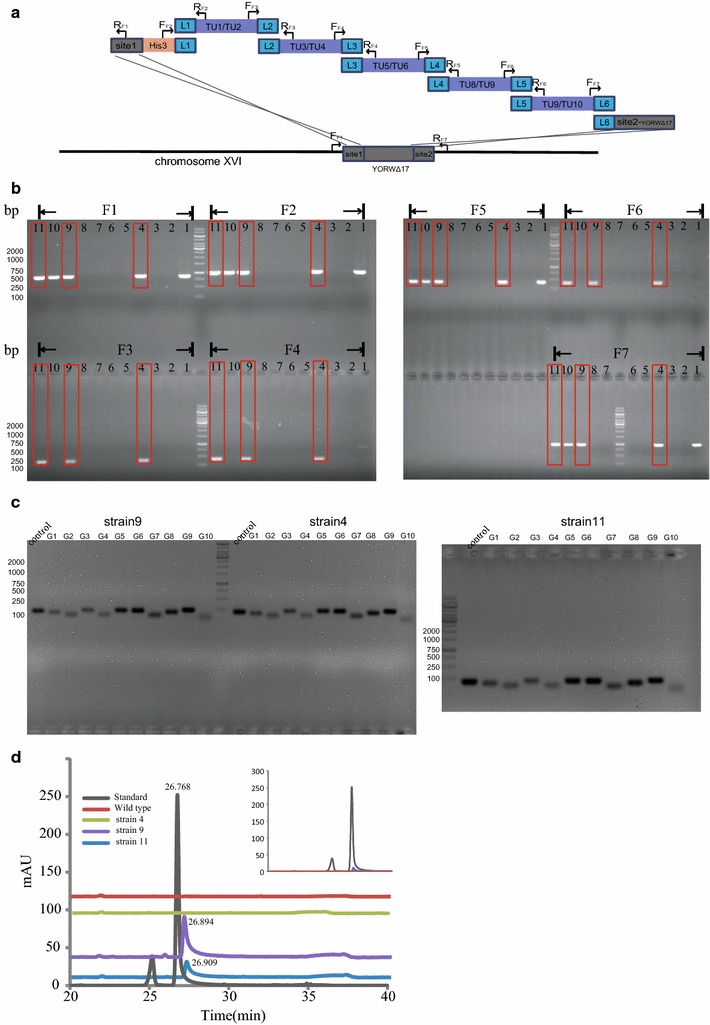
Structure and identification strategy of the assembled and integrated ten-gene pathway. **a** Structure and identification strategy of the assembled and integrated ten-gene pathway in *YORW*Δ17 site. **b** PCR gel picture of identification PCRs for assembly and integration. *F1* to *F7* indicate seven PCRs designed to confirm correct assembly and integration, *1* to *11* represent 11 strains picked from SC-His plate. Strains 4, 9, and 11 were proved to have all PCR fragments as illustrated in the *red boxes*. **c** PCR gel picture for analyzing whether all ten genes were integrated in Strains 4, 9, and 11. *G1* to *G10* indicate PCRs for gene identification. Control denotes the control gene ACT1. Negative control had no PCR bands and is not included here. **d** HPLC analysis for target product of the ten-gene biosynthetic pathway

*G7*-*pPGK1*-*pTEF2*-*G8*-*tTDH1*-L5, and L5-*tCCW12*-*G9*-*pTDH3*-*pADH1*-*G10*-*tRPL9A*-L6, were constructed with Golden Gate method as illustrated in previous text and Fig. 5a. These fragments were co-transformed with two modules, Site1-His3-L1 and L6-site2, into *S. cerevisiae* and selected on SC-His plates. Since the relevant research of this pathway was not ready to be published, furthermore, the sequence information was not relevant to the method we described in this article, all genes were represented as G1 to G10 without specific gene and sequence information.

Eleven colonies were picked for PCR identification (Fig. 5b). 7 primer sets (F_F1_ and R_F1_, F_F2_ and R_F2_, F_F3_ and R_F3_, F_F4_ and R_F4_, F_F5_ and R_F5_, F_F6_ and R_F6_, F_F7_ and R_F7_) were designed to analyze assembly and integration status on genome *YORW*Δ17 site. As shown in Fig. 5a, primers F_F1_ and R_F7_ were designed to anneal with DNA sequence flanking *YORW*Δ17 site on *chromosome XV*, R_F1_ was designed to anneal with site1 fragment, F_F2_ were designed to anneal with His3 fragment, while other primers were designed to anneal with the terminator fragments. Strains 4, 9, and 11 were identified to be assembled and integrated correctly (Fig. 5b), F1 to F7 represented corresponding PCR fragments from 7 primer sets, respectively. To further confirm that all ten genes (from G1 to G10) were included in the integration site, ten primer sets were designed to amplify within specific gene sequence, with PCR products around 100–200 bp range (Fig. 5c). Strains 4, 9, and 11 were all shown to contain the expected ten fragments. The control gene was ACT1.

Furthermore, to demonstrate the effectiveness of the ten-gene biosynthetic pathway, HPLC was used to analyze the culture of the 3 strains. Compared with standard, Strains 9 and 11 were confirmed to produce the target product (Fig. 5d).

## Conclusions

An efficient DNA assembly and chromosomal integration method was created by combining homologous recombination (HR) in *S. cerevisiae* and Golden Gate method, designated as modularized two-step (M2S) technique. Two major assembly experiments are performed consecutively to integrate multiple transcription units simultaneously as illustrated in Figs. 1 and 2. In Step 1, Modularized scaffold containing a head-to-head promoter module and a pair of terminators was assembled with two genes. Thus, two transcription units were assembled with Golden Gate method into one scaffold in one reaction (Fig. 1). In Step 2, the two transcription units were mixed with modules of selective markers and integration sites and transformed into *S. cerevisiae* for assembly and integration (Fig. 2). Modules in Step 2 were readymade, which simplified the integration procedure without frequent designing and synthesis as previous methods. By assembling most parts in Step 1 in vitro, the number of DNA cassettes for homologous integration in Step 2 was significantly reduced. Thus, high assembly efficiency, high integration capacity, and low error rate were achieved. β-carotene synthesis pathway and a ten-gene pathway were integrated to have demonstrated the advantages of this technique.

## Methods

### Strain, plasmids, and media

Trans T1 *Escherichia coli* was used as the host for plasmid construction and amplification. *S. cerevisiae* 4742 (*MATα*, *his3Δ1*, *leu2Δ0*, *lys2Δ0*, *MET15*, *ura3Δ0*) was used as the host for DNA assembly and integration. pMD-19T plasmid serve as the vectors for assembly.

Construction of P1 (pTDH3-pADH) is an example of promoter module assembly. Head-to-head promoters pTDH3-pADH with Type IIs restriction enzyme sites flanking away were assembled by overlap PCR and then inserted in plasmid pMD19T-Amp at the region between M13F and M13R (Additional file 1: Figure S1). To construct terminator Plasmids, such as T1-(tTPI1-PGIt), terminator cassette L1-tTPI1-AGGTT (adapter)- BsaI-BsaI-AATCG (adapter)- PGIt-L2 was synthesized (GENEWIZ, China) and embedded in plasmid pMD19T-Amp or pUC57-kan. For integration module plasmids in Step 2, about 500 bp sequences of the N terminal and C terminal of the integration site were used as Site1 and Site2 for homologous integration. Site1- selective marker (URA3/HIS3/LEU2)-L1 cassette was constructed by overlap PCR and then connected with pUC57-Kan vector (Additional file 1: Figure S3) for storage (Additional file 1: Table S6). Site2 was similarly constructed (Additional file 1: Table S6).

Yeast YPD medium containing 1% yeast extract, 2% peptone supplied with 2% glucose was used to cultivate *S. cerevisiae* 4742 strain. Synthetic complete drop-out medium lacking uracil (SC-Ura) was used to select transformants. During plasmid construction, Trans T1 was grown in Luria broth: 10 g/L NaCl, 10 g/L tryptone, and 5 g/L yeast extract. Ampicillin (100 mg/L) and kanamycin (50 mg/L) were used where appropriate.

### DNA assembly with Golden Gate method (Step 1)

The promoter and terminator plasmids were PCR amplified with universal primers to form corresponding modules. pTDH3-pADH1 was PCR amplified with primer set PMD-P1-FB and PMD-P1-RB, which was embedded with BsaI adapters for Golden Gate assembly. Alternatively, primer set PMD-P1-FS and PMD-P1-RS was embedded with SapI adapters for genes with native BsaI recognition sites. Similarly, terminator module was obtained by PCR amplification with T1 or T2 primer set. Coding sequences were PCR amplified with Golden Gate primers. Primers for XdCrtYB, XdCrtI, SaGGPS, and tHMG1 assembly are listed in Additional file 1: Table S5. Each fragment was mixed in equimolar amounts with 1.5 μL 10× T4 DNA ligase reaction buffer, 0.15 μL 100× bovine serum albumin, 1 μL T4 DNA ligase (2,000,000 U/mL), and 1 μL BsaI or BsmBI to a total volume of 15 μL. All reagents were purchased from New England Biolabs. The Golden Gate assembly was performed in a thermocycler as follows: 25 cycles of 37 °C for 3 min and 16 °C for 4 min, followed by 50 °C for 5 min and 80 °C for 5 min. 5 μL of assembly mixture was transformed into *E. coli* Trans T1 competent cells and incubated at 37 °C overnight on LB agar plates with Kan or Amp antibiotic.

To select correctly assembled plasmids, 10 colonies from LB agar plates with Kan or Amp antibiotic were randomly picked and boiled for PCR template. Colony PCRs were performed with 2 × Taq Master Mix (CWBiotech, China). F1 fragment was PCR amplified using the primer set L1F and L1R; F2 fragment with L2F and L3R, F3 fragment with pTDH3-F and SaGGPS-R, F4 fragment with pADH1-F and tHMG1-R, F5 fragment with pPGK1-F and CrtBY-R, and F6 fragment with pTEF2-F and CrtI-R (Fig. 3; Additional file 1: Table S5).

Selected colonies were inoculated into LB liquid medium, grown at 37 °C for 12 h, and purified with Axygen plasmid Miniprep kit for integration parts.

### Integration of modular parts (Step 2)

For the genomic integration, transcription-unit modules, selective marker, and integration homologous arm modules were transformed into *S. Cerevisiae* 4742 cells via electroporation. Competent cells were prepared according to the following protocol: Single colonies were inoculated in 4 mL liquid YPD to OD600 = 0.6–1.0; and 2 mL of cells were collected via centrifugation at 10,000*g* for 1 min. Obtained cell pellet was washed twice using 1 mL ice-cold water and then incubated in 1 mL transformation reagent (10 mM LiAc, 10 mM DTT, 0.6 M sorbitol, 10 mM pH7.5 Tris–HCl) for 20 min at 25 °C. Conditioned cells were collected by centrifugation and washed twice using 1 mL ice-cold 1 M sorbitol buffer, then resuspended to a final volume of 100 μL in sorbitol buffer. Cells with 200 or 500 ng DNA were electroporated at 2.7 kV, 25 μF, 200 Ω (Bio-Rad, Hercules, CA) and incubated in 1 mL sorbitol buffer for 1–2 h at 30 °C, then plated on selective media for 2 days.

### Measurement of β-carotene production and PCR confirmation of the correctly assembled pathways

β-carotene was extracted with a compound, that is mixed by methanol, acetonitrile, and dichloromethane (21:21:8), and was analyzed using high-performance liquid chromatography (Agilent Technologies Series 1200 system, Agilent, USA), 20 μL of extract was loaded onto the Agilent ZORBAX SB-C18 column (250 mm × 4.6 mm, 5 μm) and analyzed at 450 nm with a flow rate of 1.0 mL/min.

For PCR confirmation of transformants, single colonies were inoculated into 4 mL SC-Ura medium, then grown at 30 °C 250 rpm overnight. Cells were harvested by centrifugation; and genomic DNA was extracted by Yeast Gen DNA Kit (CWBiotech, China). 2 μL total DNA as template was PCR analyzed with 2 × Taq Master Mix (CWBiotech, China). F1 fragment was PCR amplified using the primer set L1F and L1R; F2 fragment with L2F and L3R, F3 fragment with pTDH3-F and SaGGPS-R, F4 fragment with pADH1-F and tHMG1-R, F5 fragment with pPGK1-F and CrtBY-R, F6 fragment with pTEF2-F and CrtI-R (the PCR assay result is the same as Fig. 3). To identify correct integration of β-carotene pathway, F1’ fragment was PCR amplified using the primer set F_F1’_ and R_F1’_, F2’ fragment was PCR amplified using the primer set F_F2’_ and R_F2’_. Primers F_F1’_ and R_F2’_ were designed to anneal with DNA sequence flanking *YPRC*Δ15 site on *chromosome XVI*; R_F1’_ was designed to anneal with Ura3 fragment; and F_F2’_ was designed to anneal with site2. Fragment F3’ was PCR amplified with primer set Y15site1F and Y15site2R. F4’ fragment was obtained with 15site1 (134)-F and pTDH3 (273)-R, and F5’ fragment with pTEF2 (211)-F and 15site2 (316)-R. All primers were designed be used as universal primers for verification.

PCR confirmation of TU plasmids of the 10 gene integration were similar as the 4 gene one.

## Additional file

**Additional file 1: Table S1.** The sequence of various promoter. **Table S2.** The sequence of various terminators. **Table S3.** L sequence. **Table S4.** The integration locus of chromosome. **Table S5.** PCR primers used in this work. **Table S6.** The sequence of integration locus (site2). **Figure S1.** The diagrams of promoter plasmids (Circular Display). **Figure S2.** The diagrams of terminator plasmids (linear display). **Figure S3.** The diagrams of integration locus (site1) plasmids. **Figure S4.** The transformants of β-carotene on SC-Ura solid medium.

## Abbreviations

Amp: ampicillin
Kan: kanamycin
LB: lysogeny broth
Ura: uracil
His: histidine
Leu: leucine
YPD: yeast extract peptone dextrose medium
SC-Ura: uracil synthetic drop-out media
