# Supplementary material for: Development of a modularized two-step (M2S) chromosome integration technique for integration of multiple transcription units in Saccharomyces cerevisiae
Source: Biotechnol Biofuels. 2016 Oct 28;9:232. doi: 10.1186/s13068-016-0645-4 (PMC5084435; doi:10.1186/s13068-016-0645-4)
Supplement: Supplementary file 1 — Additional file 1: Table S1. The sequence of various promoter. Table S2. The sequence of various terminators. Table S3. L sequence. Table S4. The integration locus of chromosome. Table S5. PCR primers used in this work. Table S6. The sequence of integration locus (site2). Figure S1. The diagrams of promoter plasmids (Circular Display). Figure S2. The diagrams of terminator plasmids (linear display). Figure S3. The diagrams of integration locus (site1) plasmids. Figure S4. The transformants of β-carotene on SC-Ura solid medium. [file 13068_2016_645_MOESM1_ESM.docx]

Additional file1

Table S1 the promoter library (the sequence of various promoters)

| **Promoter** | **Sequence (5′→ 3′)** |
| --- | --- |
| pTDH3 | ATACTAGCGTTGAATGTTAGCGTCAACAACAAGAAGTTTAATGACGCGGAGGCCAAGGCAAAAAGATTCCTTGATTACGTAAGGGAGTTAGAATCATTTTGAATAAAAAACACGCTTTTTCAGTTCGAGTTTATCATTATCAATACTGCCATTTCAAAGAATACGTAAATAATTAATAGTAGTGATTTTCCTAACTTTATTTAGTCAAAAAATTAGCCTTTTAATTCTGCTGTAACCCGTACATGCCCAAAATAGGGGGCGGGTTACACAGAATATATAACATCGTAGGTGTCTGGGTGAACAGTTTATTCCTGGCATCCACTAAATATAATGGAGCCCGCTTTTTAAGCTGGCATCCAGAAAAAAAAAGAATCCCAGCACCAAAATATTGTTTTCTTCACCAACCATCAGTTCATAGGTCCATTCTCTTAGCGCAACTACAGAGAACAGGGGCACAAACAGGCAAAAAACGGGCACAACCTCAATGGAGTGATGCAACCTGCCTGGAGTAAATGATGACACAAGGCAATTGACCCACGCATGTATCTATCTCATTTTCTTACACCTTCTATTACCTTCTGCTCTCTCTGATTTGGAAAAAGCTGAAAAAAAAGGTTGAAACCAGTTCCCTGAAATTATTCCCCTACTTGACTAATAAGTATATAAAGACGGTAGGTATTGATTGTAATTCTGTAAATCTATTTCTTAAACTTCTTAAATTCTACTTTTATAGTTAGTCTTTTTTTTAGTTTTAAAACACCAAGAACTTAGTTTCGAATAAACACACATAAACAAACAAA |
| pADH1 | ACATGTAGGTGGCGGAGGGGAGATATACAATAGAACAGATACCAGACAAGACATAATGGGCTAAACAAGACTACACCAATTACACTGCCTCATTGATGGTGGTACATAACGAACTAATACTGTAGCCCTAGACTTGATAGCCATCATCATATCGAAGTTTCACTACCCTTTTTCCATTTGCCATCTATTGAAGTAATAATAGGCGCATGCAACTTCTTTTCTTTTTTTTTCTTTTCTCTCTCCCCCGTTGTTGTCTCACCATATCCGCAATGACAAAAAAATGATGGAAGACACTAAAGGAAAAAATTAACGACAAAGACAGCACCAACAGATGTCGTTGTTCCAGAGCTGATGAGGGGTATCTCGAAGCACACGAAACTTTTTCCTTCCTTCATTCACGCACACTACTCTCTAATGAGCAACGGTATACGGCCTTCCTTCCAGTTACTTGAATTTGAAATAAAAAAAAGTTTGCTGTCTTGCTATCAAGTATAAATAGACCTGCAATTATTAATCTTTTGTTTCCTCGTCATTGTTCTCGTTCCCTTTCTTCCTTGTTTCTTTTTCTGCACAATATTTCAAGCTATACCAAGCATACAATCAACTATCTCATATACA |
| pPGK1 | ACGCACAGATATTATAACATCTGCACAATAGGCATTTGCAAGAATTACTCGTGAGTAAGGAAAGAGTGAGGAACTATCGCATACCTGCATTTAAAGATGCCGATTTGGGCGCGAATCCTTTATTTTGGCTTCACCCTCATACTATTATCAGGGCCAGAAAAAGGAAGTGTTTCCCTCCTTCTTGAATTGATGTTACCCTCATAAAGCACGTGGCCTCTTATCGAGAAAGAAATTACCGTCGCTCGTGATTTGTTTGCAAAAAGAACAAAACTGAAAAAACCCAGACACGCTCGACTTCCTGTCTTCCTATTGATTGCAGCTTCCAATTTCGTCACACAACAAGGTCCTAGCGACGGCTCACAGGTTTTGTAACAAGCAATCGAAGGTTCTGGAATGGCGGGAAAGGGTTTAGTACCACATGCTATGATGCCCACTGTGATCTCCAGAGCAAAGTTCGTTCGATCGTACTGTTACTCTCTCTCTTTCAAACAGAATTGTCCGAATCGTGTGACAACAACAGCCTGTTCTCACACACTCTTTTCTTCTAACCAAGGGGGTGGTTTAGTTTAGTAGAACCTCGTGAAACTTACATTTACATATATATAAACTTGCATAAATTGGTCAATGCAAGAAATACATATTTGGTCTTTTCTAATTCGTAGTTTTTCAAGTTCTTAGATGCTTTCTTTTTCTCTTTTTTACAGATCATCAAGGAAGTAATTATCTACTTTTTACAACAAATATAAAACA |
| pTEF2 | TATACTTACATATAGTAGATGTCAAGCGTAGGCGCTTCCCCTGCCGGCTGTGAGGGCGCCATAACCAAGGTATCTATAGACCGCCAATCAGCAAACTACCTCCGTACATTCATGTTGCACCCACACATTTATACACCCAGACCGCGACAAATTACCCATAAGGTTGTTTGTGACGGCGTCGTACAAGAGAACGTGGGAACTTTTTAGGCTCACCAAAAAAGAAAGAAAAAATACGAGTTGCTGACAGAAGCCTCAAGAAAAAAAAAATTCTTCTTCGACTATGCTGGAGGCAGAGATGATCGAGCCGGTAGTTAACTATATATAGCTAAATTGGTTCCATCACCTTCTTTTCTGGTGTCGCTCCTTCTAGTGCTATTTCTGGCTTTTCCTATTTTTTTTTTTCCATTTTTCTTTCTCTCTTTCTAATATATAAATTCTCTTGCATTTTCTATTTTTCTCTCTATCTATTCTACTTGTTTATTCCCTTCAAGGTTTTTTTTTAAGGAGTACTTGTTTTTAGAATATACGGTCAACGAACTATAATTAACTAAACACTAGTACC |
| pFBA | GATCCAACTGGCACCGCTGGCTTGAACAACAATACCAGCCTTCCAACTTCTGTAAATAACGGCGGTACGCCAGTGCCACCAGTACCGTTACCTTTCGGTATACCTCCTTTCCCCATGTTTCCAATGCCCTTCATGCCTCCAACGGCTACTATCACAAATCCTCATCAAGCTGACGCAAGCCCTAAGAAATGAATAACAATACTGACAGTACTAAATAATTGCCTACTTGGCTTCACATACGTTGCATACGTCGATATAGATAATAATGATAATGACAGCAGGATTATCGTAATACGTAATAGTTGAAAATCTCAAAAATGTGTGGGTCATTACGTAAATAATGATAGGAATGGGATTCTTCTATTTTTCCTTTTTCCATTCTAGCAGCCGTCGGGAAAACGTGGCATCCTCTCTTTCGGGCTCAATTGGAGTCACGCTGCCGTGAGCATCCTCTCTTTCCATATCTAACAACTGAGCACGTAACCAATGGAAAAGCATGAGCTTAGCGTTGCTCCAAAAAAGTATTGGATGGTTAATACCATTTGTCTGTTCTCTTCTGACTTTGACTCCTCAAAAAAAAAAAATCTACAATCAACAGATCGCTTCAATTACGCCCTCACAAAAACTTTTTTCCTTCTTCTTCGCCCACGTTAAATTTTATCCCTCATGTTGTCTAACGGATTTCTGCACTTGATTTATTATAAAAAGACAAAGACATAATACTTCTCTATCAATTTCAGTTATTGTTCTTCCTTGCGTTATTCTTCTGTTCTTCTTTTTCTTTTGTCATATATAACCATAACCAAGTAATACATATTCAAA |
| pHXT7 | ACTACTTCTCGTAGGAACAATTTCGGGCCCCTGCGTGTTCTTCTGAGGTTCATCTTTTACATTTGCTTCTGCTGGATAATTTTCAGAGGCAACAAGGAAAAATTAGATGGCAAAAAGTCGTCTTTCAAGGAAAAATCCCCACCATCTTTCGAGATCCCCTGTAACTTATTGGCAACTGAAAGAATGAAAAGGAGGAAAATACAAAATATACTAGAACTGAAAAAAAAAAAGTATAAATAGAGACGATATATGCCAATACTTCACAATGTTCGAATCTATTCTTCATTTGCAGCTATTGTAAAATAATAAAACATCAAGAACAAACAAGCTCAACTTGTCTTTTCTAAGAACAAAGAATAAACACAAAAACAAAAAGTTTTTTTAATTTTAATCAAAAA |
| pTPI1 | AAGGATGAGCCAAGAATAAGGGAACAAGATTTTGGTAATTTCCAAAAAATCAATAGCATGCAGGACGTTATGAAGAAGAGATCTACGTATGGTCATTTCTTCTTCAGATTCCCTCATGGAGAAAGTGCGGCAGATGTATATGACAGAGTCGCCAGTTTCCAAGAGACTTTATTCAGGCACTTCCATGATAGGCAAGAGAGAAGACCCAGAGATGTTGTTGTCCTAGTTACACATGGTATTTATTCCAGAGTATTCCTGATGAAATGGTTTAGATGGACATACGAAGAGTTTGAATCGTTTACCAATGTTCCTAACGGGAGCGTAATGGTGATGGAACTGGACGAATCCATCAATAGATACGTCCTGAGGACCGTGCTACCCAAATGGACTGATTGTGAGGGAGACCTAACTACATAGTGTTTAAAGATTACGGATATTTAACTTACTTAGAATAATGCCATTTTTTTGAGTTATAATAATCCTACGTTAGTGTGAGCGGGATTTAAACTGTGAGGACCTTAATACATTCAGACACTTCTGCGGTATCACCCTACTTATTCCCTTCGAGATTATATCTAGGAACCCATCAGGTTGGTGGAAGATTACCCGTTCTAAGACTTTTCAGCTTCCTCTATTGATGTTACACCTGGACACCCCTTTTCTGGCATCCAGTTTTTAATCTTCAGTGGCATGTGAGATTCTCCGAAATTAATTAAAGCAATCACACAATTCTCTCGGATACCACCTCGGTTGAAACTGACAGGTGGTTTGTTACGCATGCTAATGCAAAGGAGCCTATATACCTTTGGCTCGGCTGCTGTAACAGGGAATATAAAGGGCAGCATAATTTAGGAGTTTAGTGAACTTGCAACATTTACTATTTTCCCTTCTTACGTAAATATTTTTCTTTTTAATTCTAAATCAATCTTTTTCAATTTTTTGTTTGTATTCTTTTCTTGCTTAAATCTATAACTACAAAAAACACATACATAAACTAAAA |
| pTEF1 | AGTGATCCCCCACACACCATAGCTTCAAAATGTTTCTACTCCTTTTTTACTCTTCCAGATTTTCTCGGACTCCGCGCATCGCCGTACCACTTCAAAACACCCAAGCACAGCATACTAAATTTCCCCTCTTTCTTCCTCTAGGGTGTCGTTAATTACCCGTACTAAAGGTTTGGAAAAGAAAAAAGAGACCGCCTCGTTTCTTTTTCTTCGTCGAAAAAGGCAATAAAAATTTTTATCACGTTTCTTTTTCTTGAAAATTTTTTTTTTTGATTTTTTTCTCTTTCGATGACCTCCCATTGATATTTAAGTTAATAAACGGTCTTCAATTTCTCAAGTTTCAGTTTCATTTTTCTTGTTCTATTACAACTTTTTTTACTTCTTGCTCATTAGAAAGAAAGCATAGCAATCTAATCTAAGTTTTAATTACAAA |
| pPDC1 | CATGCGACTGGGTGAGCATATGTTCCGCTGATGTGATGTGCAAGATAAACAAGCAAGGCAGAAACTAACTTCTTCTTCATGTAATAAACACACCCCGCGTTTATTTACCTATCTCTAAACTTCAACACCTTATATCATAACTAATATTTCTTGAGATAAGCACACTGCACCCATACCTTCCTTAAAAACGTAGCTTCCAGTTTTTGGTGGTTCCGGCTTCCTTCCCGATTCCGCCCGCTAAACGCATATTTTTGTTGCCTGGTGGCATTTGCAAAATGCATAACCTATGCATTTAAAAGATTATGTATGCTCTTCTGACTTTTCGTGTGATGAGGCTCGTGGAAAAAATGAATAATTTATGAATTTGAGAACAATTTTGTGTTGTTACGGTATTTTACTATGGAATAATCAATCAATTGAGGATTTTATGCAAATATCGTTTGAATATTTTTCCGACCCTTTGAGTACTTTTCTTCATAATTGCATAATATTGTCCGCTGCCCCTTTTTCTGTTAGACGGTGTCTTGATCTACTTGCTATCGTTCAACACCACCTTATTTTCTAACTATTTTTTTTTTAGCTCATTTGAATCAGCTTATGGTGATGGCACATTTTTGCATAAACCTAGCTGTCCTCGTTGAACATAGGAAAAAAAAATATATAAACAAGGCTCTTTCACTCTCCTTGCAATCAGATTTGGGTTTGTTCCCTTTATTTTCATATTTCTTGTCATATTCCTTTCTCAATTATTATTTTCTACTCATAACCTCACGCAAAATAACACAGTCAAATCAATCAAA |
| pPYK1 | AATGCTACTATTTTGGAGATTAATCTCAGTACAAAACAATATTAAAAAGAGGTGAATTATTTTTCCCCCCTTATTTTTTTTTTGTTAAAATTGATCCAAATGTAAATAAACAATCACAAGGAAAAAAAAAAAAAAAAAAAAAATAGCCGCCATGACCCCGGATCGTCGGTTGTGATACGGTCAGGGTAGCGCCCTGGTCAAACTTCAGAACTAAAAAAATAATAAGGAAGAAAAAAATAGCTAATTTTTCCGGCAGAAAGATTTTCGCTACCCGAAAGTTTTTCCGGCAAGCTAAATGGAAAAAGGAAAGATTATTGAAAGAGAAAGAAAGAAAAAAAAAAAATGTACACCCAGACATCGGGCTTCCACAATTTCGGCTCTATTGTTTTCCATCTCTCGCAACGGCGGGATTCCTCTATGGCGTGTGATGTCTGTATCTGTTACTTAATCCAGAAACTGGCACTTGACCCAACTCTGCCACGTGGGTCGTTTTGCCATCGACAGATTGGGAGATTTTCATAGTAGAATTCAGCATGATAGCTACGTAAATGTGTTCCGCACCGTCACAAAGTGTTTTCTACTGTTCTTTCTTCTTTCGTTCATTCAGTTGAGTTGAGTGAGTGCTTTGTTCAATGGATCTTAGCTAAAATGCATATTTTTTCTCTTGGTAAATGAATGCTTGTGATGTCTTCCAAGTGATTTCCTTTCCTTCCCATATGATGCTAGGTACCTTTAGTGTCTTCCTAAAAAAAAAAAAAGGCTCGCCATCAAAACGATATTCGTTGGCTTTTTTTTCTGAATTATAAATACTCTTTGGTAACTTTTCATTTCCAAGAACCTCTTTTTTCCAGTTATATCATGGTCCCCTTTCAAAGTTATTCTCTACTCTTTTTCATATTCATTCTTTTTCATCCTTTGGTTTTTTATTCTTAACTTGTTTATTATTCTCTCTTGTTTCTATTTACAAGACACCAATCAAAACAAATAAAACATCATCACA |

Table S2 terminator library (the sequence of various terminators)

| **Terminator** | **Sequence (5′→ 3′)** |
| --- | --- |
| TPI1t | GATTAATATAATTATATAAAAATATTATCTTCTTTTCTTTATATCTAGTGTTATGTAAAATAAATTGATGACTACGGAAAGCTTTTTTATATTGTTTCTTTTTCATTCTGAGCCACTTAAATTTCGTGAATGTTCTTGTAAGGGACGGTAGATTTACAAGTGATACAACAAAAAGCAAGGCGCTTTTTCTAATAAAAAGAAGAAAAGCATTTAACAATTGAACACCTCTATATCAACGAAGAATATTACTTTGTCTCTAAATCCTTGTAAAATGTGTACGATCTCTATATGGGTTACTCATAAGTGTACCGAAGACTGCATTGAAAGTTTATGTTTTTTCACTGGAGGCGTCATTTTCGCGTTGAGAAGATGTTCTTATCCAAATTTCAACTGTTATATAG |
| PGIt | AACAAATCGCTCTTAAATATATACCTAAAGAACATTAAAGCTATATTATAAGCAAAGATACGTAAATTTTGCTTATATTATTATACACATATCATATTTCTATATTTTTAAGATTTGGTTATATAATGTACGTAATGCAAAGGAAATAAATTTTATACATTATTGAACAGCGTCCAAGTAACTACATTATGTGCACTAATAGTTTAGCGTCGTGAAGACTTTATTGTGTCGCGAAAAGTAAAAATTTTAAAAATTAGAGCACCTTGAACTTGCGAAAAAGGTTCTCATCAACTGTTTAAAAGGAGGATATCAGGTCCTATTTCTGACAAACAATATACAAATTTAGTTTCAAAGATGAATCAGTGCGCGAAGGACATAACTCATGAAGCCTCCAGTATACC |
| ADH1t | AGTTATAAAAAAAATAAGTGTATACAAATTTTAAAGTGACTCTTAGGTTTTAAAACGAAAATTCTTATTCTTGAGTAACTCTTTCCTGTAGGTCAGGTTGCTTTCTCAGGTATAGCATGAGGTCGCTCTTATTGACCACACCTCTACCGGCATGCCGA |
| CYC1t | TCATGTAATTAGTTATGTCACGCTTACATTCACGCCCTCCCCCCACATCCGCTCTAACCGAAAAGGAAGGAGTTAGACAACCTGAAGTCTAGGTCCCTATTTATTTTTTTATAGTTATGTTAGTATTAAGAACGTTATTTATATTTCAAATTTTTCTTTTTTTTCTGTACAGACGCGTGTACGCATGTAACATTATACTGAAAACCTTGCTTGAGAAGGTTTTGGGACGCTCGAAGGCTTTAATTTGC |
| tFBA1 | GTTAATTCAAATTAATTGATATAGTTTTTTAATGAGTATTGAATCTGTTTAGAAATAATGGAATATTATTTTTATTTATTTATTTATATTATTGGTCGGCTCTTTTCTTCTGAAGGTCAATGACAAAATGATATGAAGGAAATAATGATTTCTAAAATTTTACAACGTAAGATATTTTTACAAAAGCCTAGCTCATCTTTTGTCATGCACTATTTTACTCACGCTTGAAATTAACGGCCAGTCCACTGCGGAGTCATTTCAAAGTCATCCTAATCGATCTATCGTTTTTGATAGC |
| tPDC1 | GCGATTTAATCTCTAATTATTAGTTAAAGTTTTATAAGCATTTTTATGTAACGAAAAATAAATTGGTTCATATTATTACTGCACTGTCACTTACCATGGAAAGACCAGACAAGAAGTTGCCGACAGTCTGTTGAATTGGCCTGGTTAGGCTTAAGTCTGGGTCCGCTTCTTTACAAATTTGGAGAATTTCTCTTAAACGATATGTATATTCTTTTCGTTGGAAAAGATGTCTTCCAAAAAAAAAACCGATGAATTAGTGGAACCAAGGAAAAAAAAAGAGGTATCCTTGATTAAGGAACA |
| tRPS2 | GCTTGTTGTCTACAAATTATAAAATAGTTTTTTATAATCAACCATTAAATAATTTTGTACACTTATAATAACACTTTATTTCTCGTTCCAATTATGCGATGCATGTGCCTTCCGTTTAGAGTGAGCATTATCAAATATAAAGTCAAATCCTTTTGGCATTTATTTTGATTGAAACCCAGTCTGTCCTTAACTCTTTTGCCTATCAAACGCTGTAGGTCACTCTTGGTACGGTACAGCGGATAGCGCTATTTATTCAGCAAGAGAAGCAAGATTAGGCGACTACAATAGATCAAAATTTTA |
| tTDH1 | ATAAAGCAATCTTGATGAGGATAATGATTTTTTTTTGAATATACATAAATACTACCGTTTTTCTGCTAGATTTTGTGAAGACGTAAATAAGTACATATTACTTTTTAAGCCAAGACAAGATTAAGCATTAACTTTACCCTTTTCTCTTCTAAGTTTCAATACTAGTTATCACTGTTTAAAAGTTATGGCGAGAACGTCGGCGGTTAAAATATATTACCCTGAACGTGGTGAATTGAAGTTCTAGGATGGTTTAAAGATTTTTCCTTTTTGGGAAATAAGTAAACAATATATTGCTGCCTT |
| tCCW12 | ACTTAGTTTATTATTATTTATACATTCTAAATTTTTTATAAACTTTTTTGGCATTTAACAAATATATAACAATTGGGATTTAGTAGAAATTCTTTTAATTTCTTCTTTTTTTTTTTAATCTATATTTATTCTTTTTCTTTAATTTTGTTCGAAACGCATATAATGTTTTTATTGATTTTGTTTCAGGTTCCTGAACCTCGCGGAGTTCCTATTTTACTGTTGTTTTTTTTCAAAACACAGGCGTACCTTGAAGTATACGCAGTGCCTCTTAGCCAAGGATAATATAAACACACTAGAACA |
| tRPL9A | GCTTTTTATTTGATTTTGTGACATTTTCTTTAAAGTAGCAGAAAAAATAATTACATATAAAAAACTTTGTATATCTAACATATAATATAATTGAACCGATACGAAAATAAAAGTACCGATAATATGTCAACTTTTTGTTTTTTGTTGCCGTTTTATTACGTGCACTAATACATATACATGCGTATATAAATATACATAACTCTTGAGGGGTTTTGTTTCTATCT |

Table S3 L sequence (homologous sequences among transcription units)

| **L sequence** | **Sequence (5′→ 3′)** |
| --- | --- |
| **L1** | CCCCGGTCCGTTTGTTCTATACTTCTCTCTGCTATACCTACAAGCAAGGTAATCGGAAGTAGTATTACGCAGGAATATCCCGCGCGAAGCTACAATTTTTGGACTCCAACGTCAAAGCAGGGGAGTCAGAAGTCCCCTCTAAAATTGCCT |
| **L2** | GACAAAGCGCCAAGGAACTGTAATATATAGCTACGCCCTATCTGGACGATTGGGCGACTTTTACGTACGGTTGCTCAATTCCTACGCAACTTAATATATTTTGCAACGGTTAAATCGGCTTGAAGCTCGGGCTATCCAACTCGCGGACTA |
| **L3** | AACGACGGTAGACGCCAACTACGCTGACAGACCGATTTGTTTAAGATTAGAAGATTTTTAGCCGCGCCGCAATCGGAACCAGCAAACTCAATTCTGGGAACAGTTTAAAATACTAGTAATTACGATAGCCGAGAAACGGACTAAGTCCGC |
| **L4** | CCAGACGATACAGAGGCTAAGAATAACGCAGATAATCGCTCTAACGAAACGTACTAAAAGATTTCTTTTGAAGTAACTAGATACCCTGGTCTTATACTAGGTATCTTTGTCAGAAACGGCCTAAGACTACAGTAAGAGCAGTTGGAACCT |
| **L5** | CAGCCAACCAGTCAGATTAGCAGTATATAGGCTGCGAGTTACTAGCGGGCAAAATCTGGAGTTATCGCCTAAAGCGTTATCTGATCCCTATTCGGCAGCAGTTTGGTCGGTTTGAGTAGTCAATAACGTTCTATCGTATCTCGTTCGTCG |
| **L6** | ATTGGACGAGTTCTACCTGACAGAACGGGGCTGGGAAGAGAAAGATCCTAGCTGGCGCCTATACTACTTCCAAGAACCATTCCCCAGCTTATCAAGAGTTTTCTCGTTCAGCTACCGACTGTATTGAGTTGGAGACAAGGCGTATCGGAA |

Table S4 the integration locus of chromosome

| **locus** | **Description** |
| --- | --- |
| 15site | Saccharomyces cerevisiae S288c chromosome XVI, long_terminal_repeat and Autonomously Replicating Sequence (775918-776796) |
| 17site | Saccharomyces cerevisiae S288c chromosome XV, long_terminal_repeat ( 664035-665683) |
| 22site | Saccharomyces cerevisiae S288c chromosome XV, long_terminal_repeat (969471-970123) |

Table S5 PCR primers used in this work

| **Primers** | **Sequence (5′→ 3′)** | **Sources** |
| --- | --- | --- |
| PMD-P1-FB | CTCTGGTCTCACATCTTTGTTTGTTTATGTG | This study |
| PMD-P1-FB | CTCTGGTCTCAATTCTGTATATGAGATAGTTG | This study |
| T1-FB | CTCGGTCTCAATCGAACAAATCGCTCTTAA | This study |
| T1-RB | GAATTCGGTCTCAACCTGATTAAT | This study |
| P2-Fs | CCTGCTCTTCCATCTGTTTTATATTTGTTGTAA |  |
| P2-Rs | GCAGCTCTTCATTCGGTACTAGTGTTTAGTTAA |  |
| T2-Fs | CTCGCTCTTCATCGAACAAATCGCTCTTAAATATATAC | This study |
| T2-Rs | CCAGCTCTTCACCTGATTAATATAATTATATAAAAATATTATCTTCTTTTC | This study |
| L1-F | CGTCTCCCCCGGTCCGTTTG | This study |
| L2-R | GTCCGCGAGTTGGATAGCCCG | This study |
| L2-F | CGACAAAGCGCCAAGGAACTG | This study |
| L3-R | GCGGACTTAGTCCGTTTCTCGG | This study |
| Y15site1F | GCCAGGCGCCTTTATATCATATAATTAAGACA | This study |
| Y15site1-URA3R (L1R) | CTCGAGAGGCAATTTTAGAGGGGACTTC | This study |
| Y15site2F (L3F) | CGACGGTAGACGCCAACTACGC | This study |
| Y15site2R | ATAAAGCAGCCGCTACCAAACAG | This study |
| XdCrtYB-F | CCTGCTCTTCAGATATGACGGCTCTCGCATATTACC | This study |
| XdCrtYB-R | CCAGCTCTTCAAGGTTACTGCCCTTCCCATCCG | This study |
| XdCrtI-F | CCAGCTCTTCTGAAATGGGAAAAGAACAAGATCAGGA | This study |
| XdCrtI-R | CCTGCTCTTCACGATCAGAAAGCAAGAACACCAACG | This study |
| tHMG1-S | CCAGGTCTCAGAATAAAACAATGGCTGCAGACCAAT | This study |
| tHMG1-R | CCAGGTCTCTCGATTTAGGATTTAATGCAGGTGACGG | This study |
| SaGGPS-F | CCAGGTCTCAGATGATGTCATACTTCGATAACTACTT | This study |
| SaGGPS-R | CCAGGTCTCAAGGTTTATTTTCTTCTTCTGATAGTGA | This study |
| YO17-site1-F | TGTGCACAAAGGCCATAATATTATGTCTAC | This study |
| YO17-site1-R | AGGCAATTTTAGAGGGGACTTCTGACT | This study |
| YO17-site2-F | CGACGGTAGACGCCAACTACGC | This study |
| YO17-site2-R | AAAGCTGGCTCCCCTTAGACAAATACGC | This study |
| pTDH3-F | CTAGCGTTGAATGTTAGCGTC | This study |
| pADH1-F | CATGTAGGTGGCGGAGGGGAG | This study |
| pPGK1-F | CGCACAGATATTATAACATCTG | This study |
| pTEF2-F | CTTACATATAGTAGATGTCAAG | This study |
| 15site1(134)-F | GTCAGATGAATGGACGCGAATG | This study |
| pTDH3(273)-R | ATTGACCCACGCATGTATCTATCTC | This study |
| pTEF2 (211) –F | CTGGTGTCGCTCCTTCTAGTGC | This study |
| 15site2(316)-R | CCAATTAATCACAAGTTGGTAATGAG | This study |
| F_F1’_ | GATTGTTGGCAAAGACTATAATATTATGC | This study |
| R_F1’_ | CATGTTTCTTCAACACTACATATGCG | This study |
| F_F2’_ | CGATTGTTGGCAAAGACTATAATATTATGC | This study |
| R_F2’_ | GGGAAAAAAATGATGAAGAGTAATGCC | This study |
| FF1 | ATTTCGCCCAGGATCGAACTG | This study |
| RF1 | TCCTCGAGAATATGGGAATGCAC | This study |
| FF2 | GGAGATCTCTCTTGCGAGATGATCC | This study |
| RF2 | GCTCGAATTCGGTCTCAACCTG | This study |
| FF3 | GTGCGCGAAGGACATAACTC | This study |
| RF3 | ACCACACCTCTACCGGCATG | This study |
| FF4 | TTTGGGACGCTCGAAGGC | This study |
| RF4 | ATTAACGGCCAGTCCACTGC | This study |
| FF5 | CCGATGAATTAGTGGAACCAAGG | This study |
| RF5 | AGCAAGATTAGGCGACTACAATAGATC | This study |
| FF6 | GTAAACAATATATTGCTGCCTTGAGC | This study |
| RF6 | GCAGTGCCTCTTAGCCAAGG | This study |
| FF7 | TTACGTGCACTAATACATATACATGCG | This study |
| RF7 | GAGCATTTCGTTCACTTACCAAAC | This study |

Table S6 the sequence of integration locus (site2)

| **Site2** | **Sequence (5′→ 3′)** |
| --- | --- |
| 15site2 | AATGGAAGGTCGGGATGAGCATATACAAGCACTAAGAAGAACAATACAGAACTCTACACGGTATTATTGTGCTACAAGCTCGAGTAAAACCGAGTGTTTTGACGATACTAACGTTGTTAAGAAAGTAACTTGTTATCAAACTCATTACCAACTTGTGATTAATTGGTGAATAATATGATAATTGTCGAAATTCCATTGTTGGTAAAGCCTATAATATTATGTATACAGATTATACTAGAAATTCTCTCGAGAATATAAGAATCCCCAAAATTGAATCGGTATTTCTACATACTAATATTACCATTACTTCTCCTTTCGTTTTATATGTTTCATTCCTATTACATTATCGATCTTTGCATTTCAGCTTCCATTATATTTGATGTCTGTTTTATGTCCCCACGTTACACCGCATGTGACAGTATACTAGTAACATGAGTGCTACCGAATAGATGACATTTTAGACTTTCATTCCAACAACTTGGTTGACAGAATGTTACGTACCCTATATCTAATCTATATGAGGCCTGAATCTAACTGAAAGGTGGAATTTCAGTAATTTATCAAGCTTTAATAAGTTTGGGTAGTTTAACTGTGCAAAAAGGTATTTACCTTACATACTGAATCTTGTCTGTTTGGTAGCGGCTGCTTTAT |
| 17site2 | AATCGTCCCCAACAAAAGTGGGCTCTCAAAATTCATCACATTTAAATGCATATAGGAAGAGCAACAGTTGGTTTGCATCTGATGTTCCTTAAAGATTTCGACATAATGTGCGAAGTAGATAAAATGGGTCATTTATTAATAGTTATTTCATTATTAACCAGTTGTGGTACAAATGCAACTAAAGAAAAAAACTACTAAACTATCCGGGAAATGCGCCTTAGATTGCACTTCTTAATTCTTATTTTCGATTTTTATTTTTCCTTTGATAATCATAAAGAGAAACGACGATCATTTCTAAAGCCATTTCTGCTAGTATACCGTTAAATAAGAAAAATAAAGCCAAATATTATAATTTTTCTAATGTGAATCCATAAATATCAAAGCATGCAAAAAGGGAAAGAAGTAATGTCTTGGATTTATATAGCGTATTTGTCTAAGGGGAGCCAGCTTT |
| 22site2 | GGACCAACTATCATCCGCTAATTACTGACATTACCAAATGAGATCTGTGAATGGGCAAGATAAAAAACAAAAATTGAAATGTTTGACGTTATGTAAAACTATTAATTCCTTCGCTTTCGGCGGTCACAGAATTTGCGTGTAGCTGACTCTTGTTCAATCAATATCATTTGTTACTTTATTTGAAAGTCTGTATTACTGCGCCTATTGTCATCCGTACCAAAGAACGTCAAAAAGAAACAAGATAATTTTTGTGCTTACACCATTTATAGATCACTGAGCCCAGAATATCGCTGGAGCTCAGTGTAAGTGGCATGAACACAACTCTGACTGATCGCACATATTGCCGTTATCATAAATACTAGTTGTACTTGTCAATGCGACGAATGGCATCATGCCTATTATTACGTTCCTCTTTTTCCGTTTCATGTTTCCAGAATGCTATTGAATCTAACACTTCAATTATAAAAAAGAATAAATCCGCAATAATTTTAGGCTAATTGTTGTACTGTCAAGCGAACCTAATGGTTAAAATTCAGAGGAACCTTCGACGTAGTCTGATCGCTACTTCTATATCTTATGTTCCCAGTCAATCAAAAGTTGATACTATAATAGCTGCCATTTATACCTGTTAGTTATGGCGATCGTTTATCACG |

Figure S1 the diagrams of promoter plasmids(Circular Display)


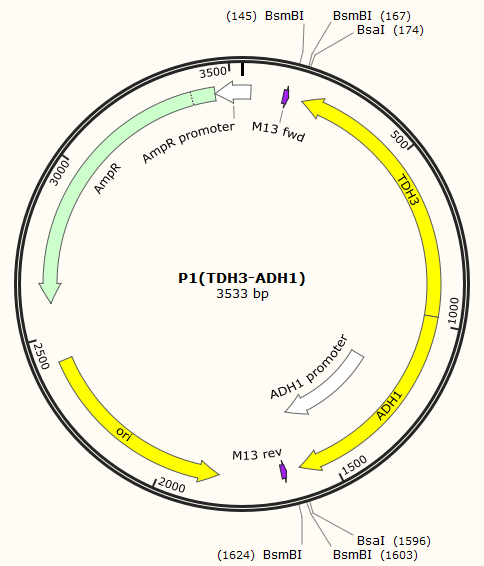


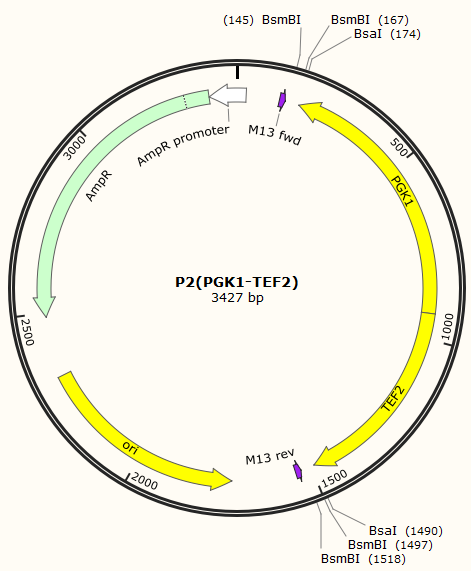


Figure S2 the diagrams of terminator plasmids (linear display)


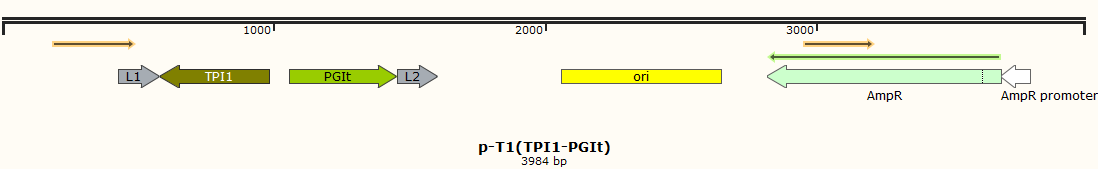


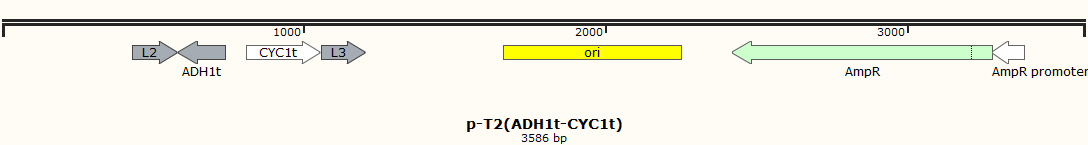


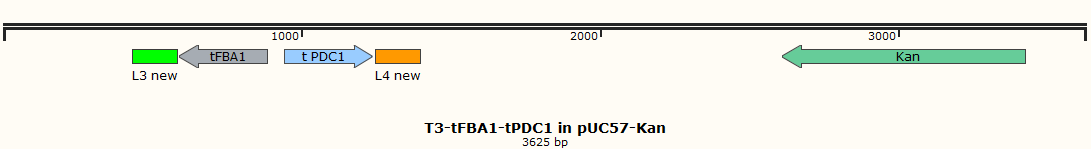


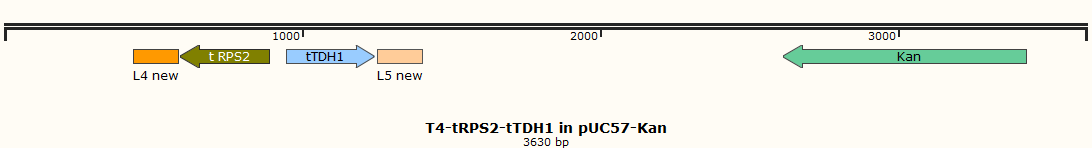


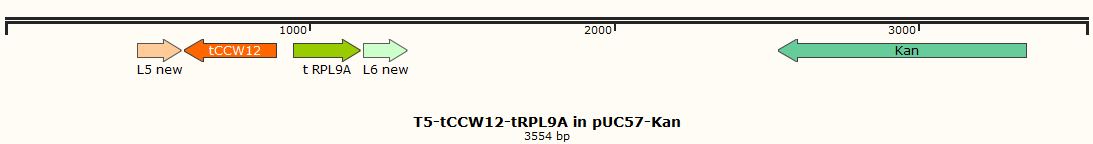


Figure S3 the diagrams of integration locus (site1) plasmids


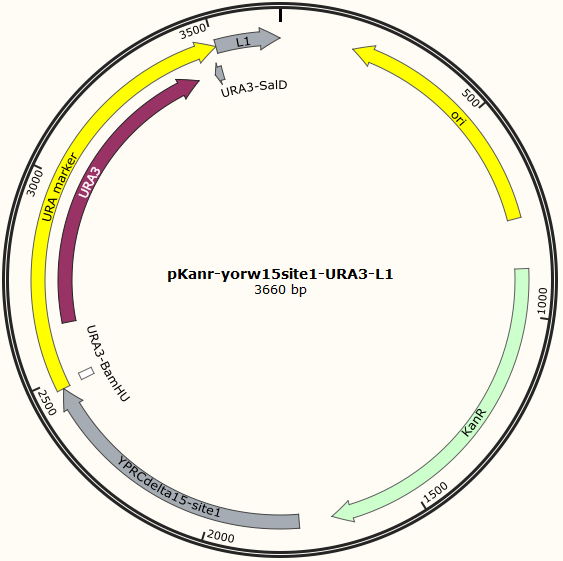


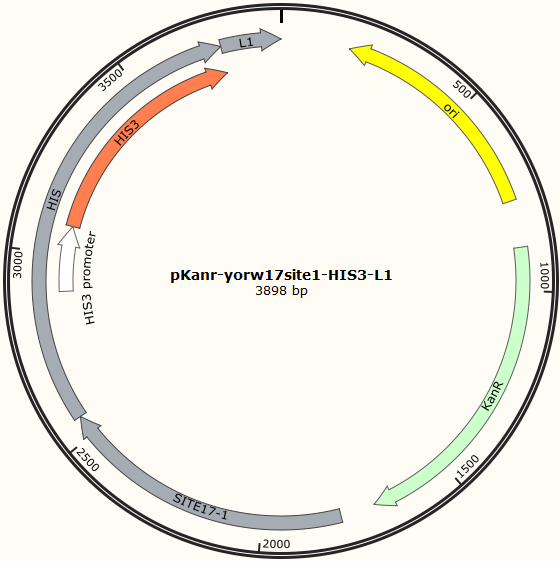


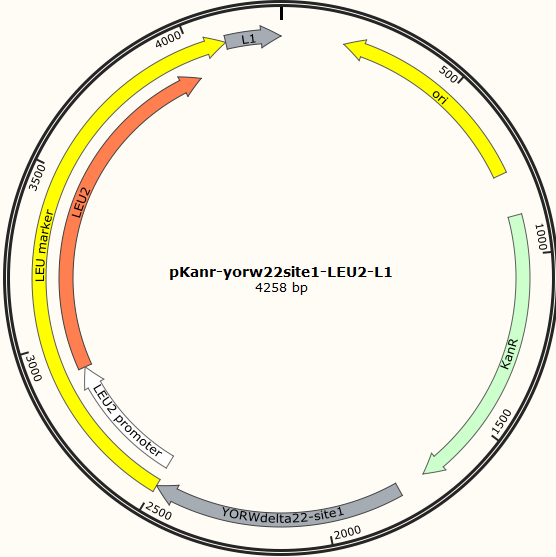


Figure S4 Resulting S. cerevisiae transformants from Step 2 assembly on SC-Ura solid medium. Orange colonies were confirmed to contain correctly assembled β-[carotene](http://cn.bing.com/dict/search?q=carotene&FORM=BDVSP6&mkt=zh-cn) synthetic genes and able to produce β-[carotene](http://cn.bing.com/dict/search?q=carotene&FORM=BDVSP6&mkt=zh-cn) ( Electroporation with 200 ng DNA and 500 ng DNA).


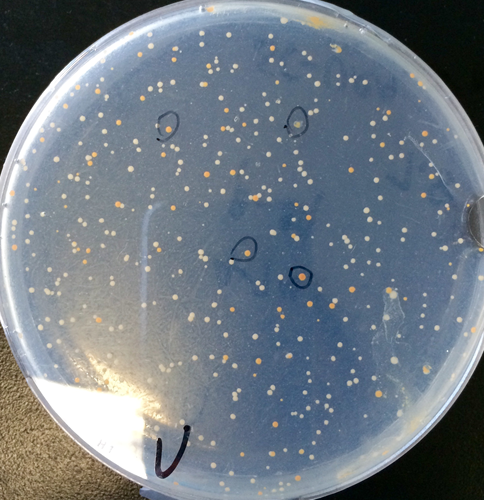


**
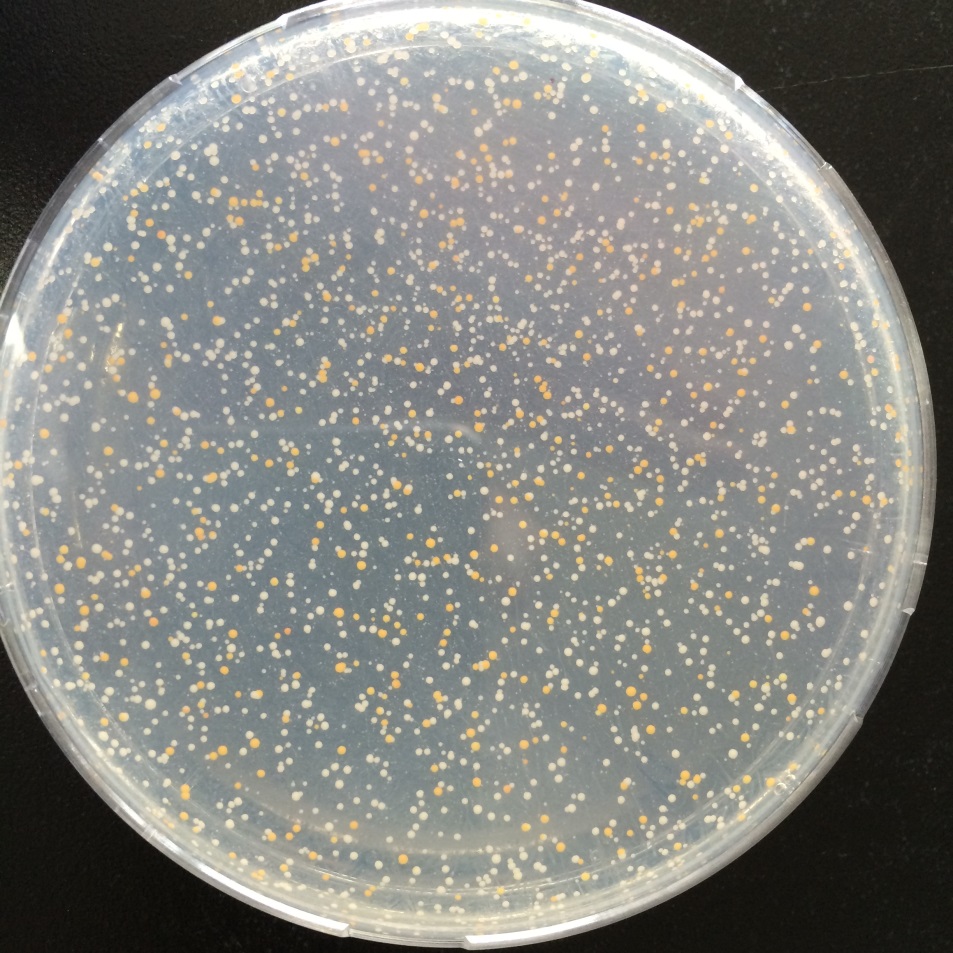
**
